# Corrigendum to “Citation analysis of scientific categories” [Heliyon 3 (5) (May 2017) e00300]

**DOI:** 10.1016/j.heliyon.2019.e01243

**Published:** 2019-03-20

**Authors:** Gregory S. Patience, Christian A. Patience, Bruno Blais, Francois Bertrand

**Affiliations:** a Department of Chemical Engineering, Polytechnique Montréal, C.P. 6079, Succ. CV, Montréal, H3C 3A7 Québec, Canada; b Department of Mechanical Engineering, McGill University, Montréal, Québec, Canada

In the Excel file of the supplementary content “Top 10 Journals per Scientific Category”, the category “Materials Science Coating Films” only has 7 journals but the calculations in this file included 10. So, all the attributions from line 1114 downwards are displaced by 3 lines. That is, the three top journals from “Dermatology” are included in “Materials Science Coating Films”, then three journals from “Political Science” are in “Dermatology”, etc. The authors apologize for the mistake. A corrected supplementary file is displayed below. This correction does not, in any way, compromise the findings of the study, either in terms of the methodology, results, or interpretations drawn from the data therein.

## Additional information

Supplementary content related to this article has been published online at https://doi.org/10.1016/j.heliyon.2019.e01243.

No additional information is available for this paper.

### Supplementary material

The following Supplementary material is associated with this article:

Table S1 Top 10 journals per category. Table S1

